# An engaged approach to exploring issues around poverty and mental health: A reflective evaluation of the research process from researchers and community partners involved in the DeStress study

**DOI:** 10.1111/hex.13065

**Published:** 2020-05-24

**Authors:** Felicity Thomas, Lorraine Hansford, Katrina Wyatt, Richard Byng, Karen Coombes, Jenna Finch, Kirsty Finnerty, Joe Ford, Keith Guppy, Rachel Guppy, Susanne Hughes, Rose McCabe, Hilary Richardson, Debbie Roche, Hazel Stuteley

**Affiliations:** ^1^ University of Exeter Exeter UK; ^2^ University of Plymouth Plymouth UK; ^3^ City University of London London UK

**Keywords:** engagement, health inequalities, health policy, mental health, patient and public involvement, poverty, research design, socio‐economic factors

## Abstract

**Background:**

Involving patients, service users, carers and members of the public in research has been part of health policy and practice in the UK for the last 15 years. However, low‐income communities tend to remain marginalized from the co‐design and delivery of mental health research, perpetuating the potential for health inequalities. Greater understanding is therefore needed on how to meaningfully engage low‐income communities in mental health research.

**Objectives:**

To explore and articulate whether and how an engaged research approach facilitated knowledge coproduction relating to poverty and mental distress.

**Setting:**

A reflective evaluation of community and researcher engagement in the DeStress study that took place in two low‐income areas of South‐west England.

**Design:**

Reflective evaluation by the authors through on‐going feedback, a focus group and first‐person writing and discussion on experiences of working with the DeStress project, and how knowledge coproduction was influenced by an engaged research approach.

**Results:**

An engaged research approach influenced the process and delivery of the DeStress project, creating a space where community partners felt empowered to coproduce knowledge relating to poverty‐related mental distress, treatment and the training of health professionals that would otherwise have been missed. We examine motivations for involvement, factors sustaining engagement, how coproduction influenced research analysis, findings and dissemination of outputs, and what involvement meant for different stakeholders.

**Conclusion:**

Engaged research supported the coproduction of knowledge in mental health research with low‐income communities which led to multiple impacts.

## 1 BACKGROUND

It is widely recognized that patient and public involvement and engagement in health‐ and social care research leads to more relevant research, widening participation and increased involvement in service design and delivery.^1^, ^2^, ^3^, ^4^ An engaged research approach therefore is one which develops partnerships with people with experience of the issue to explore an area of mutual interest and co‐create new knowledge to inform practice.^5^, ^6^

A growing body of literature within the field of mental health focuses on survivor and service‐user‐led research and the related field of ‘mad studies’,^7^, ^8^ including the role of service‐user researchers who bring both academic training and lived experience of mental health services. Within this literature, a number of approaches to patient engagement now exist, reflecting diverse disciplinary origins, as well as commonalities in their underpinning philosophies and potential for learning.^9^ Coproduction of knowledge through engaged research has clear parallels with participatory action research and community‐based participatory research, particularly in the ways that trust, collaboration, and shared decision making and ownership are sought.^10^, ^11^

These democratic approaches which have their roots in emancipatory research are concerned with service users directly influencing the research process.^12^ They aim to shift away from professional knowledge and control towards developing partnerships which can challenge assumptions and systems to address the rights and aspirations of those affected by the issue at hand.^13^ Such approaches thus recognize the potential for evidence and knowledge to take a range of transdisciplinary and, non‐hierarchical forms, have increased reflexivity and be coproduced outside of the tight and often limited bounds of academia.^10^, ^14^

However, barriers remain in realizing coproduction in which knowledge and evidence that come from beyond academia are genuinely valued within mental health understanding and decision making. Rose and Kalathil^15^ argue that coproduction between professionals and service users will remain a fundamentally unequal relationship as long as the experiential knowledge that is brought to the relationship is defined by the expert knowledge of the professional, and when forms of marginalization within public and academic institutions become replicated within mental health research. Much of the focus here has been on the ways in which professionals maintain control over spaces of user involvement^16^ and the potential for these spaces to perpetuate white privilege by silencing Black and minority ethnic voices and experience.^14^, ^17^

In this paper, we focus on the under‐representation of people from low‐income backgrounds in mental health research, either as partners or as participants.^18^ This means that there is a paucity of understanding regarding the intersection between poverty and mental health and the perceived relevance of mental health diagnoses and treatment for those affected by poverty‐related distress. For example, the prescribing of anti‐depressants has risen dramatically in the UK, particularly in areas of economic disadvantage^19^, ^20^—however, making mental health services and interventions available does not necessarily mean that they are appropriate, or will be universally accessed. Indeed, it is argued that a bio‐medical model that frames poverty‐related mental distress as a pathological problem of the self that can be ‘corrected’ through medical or therapeutic intervention^21^ may reinforce individualized notions of blame and responsibility and mask the root causes of deprivation and social injustice known to erode well‐being.^22^, ^23^ Hence, the possibility of a conceptually flawed model of mental distress, and the need to better support the mental health of low‐income communities makes the engagement of individuals affected by these issues critical in any re‐think.

### 1.1 The DeStress study

The DeStress study sought to explore how poverty‐related mental distress is conceptualized and responded to by people living in areas of economic disadvantage and by health professionals seeking to support them. Undertaken by an interdisciplinary research team using a mixed‐methods approach (see ^18^ for further detail), DeStress was undertaken over a period of thirty months in two sites in South‐west England representing the most deprived quintile of the overall Index of Multiple Deprivation 2015.

DeStress sought to actively address the under‐representation of low‐income communities in mental health research by developing a collaborative approach to designing and delivering the research so that it was responsive to the priorities of these communities from the outset. Underpinning the development of collaborative partnerships between residents and researchers was the Connecting Communities (C2) approach, which seeks to create the conditions for health and well‐being in low‐income communities through transformative community engagement.^24^ Trust, reciprocity and active listening are considered to be necessary qualities for building relationships to co‐create services and research.^25^

Oversight of the project was achieved through a project Advisory Board. Membership included community partners, health practitioners, and representatives from public and mental health organizations. Early discussions within the study sites found a preference for membership to be open to any interested community members. This created flexibility for people to dip in and out depending on other commitments and enabled inclusivity across the different neighbourhoods within the study sites.

Eight residents from the study sites chose to sit on the Advisory Board. Whilst this met formally on five occasions during the thirty‐month project, the involvement of a Community Connector facilitated engagement between researchers and community partners throughout the course of the project, enabling a collaborative approach to shaping the research questions and methodology, interpreting study data, developing training resources for health professionals and disseminating project findings.

Bringing together the reflections of the community partners and the research team, this co‐authored paper explores whether and how the engaged research approach used facilitated knowledge coproduction relating to poverty and mental distress. We focus on how spaces were created so that people from low‐income communities (the community partners) could contribute to knowledge production; and whether and how power relations and imbalances between academics and low‐income residents could be addressed. In so doing, we acknowledge the challenges and tensions inherent within an engaged research process in order to share our learning about creating the right conditions for engagement and knowledge coproduction.

## 2 METHODS

This paper provides a reflective evaluation of the engaged approach used within DeStress from the perspectives of the research team (n = 7) and community partners (n = 8) who co‐author this paper. This was undertaken via regular verbal feedback elicited from and amongst community partners and researchers, a reflective focus group, and first‐person writing/discussion. Verbal feedback (written up as notes) was gathered via the project's Community Connector, Susanne, who was employed by the University and already known and trusted within the study sites. Susanne was in frequent and regular (at least weekly) face‐to‐face or telephone contact with community partners and researchers throughout the course of the project, acting as a conduit for reflection on the way the project was being run and advising on changes needed to ensure that community partners felt able to participate in a way that was meaningful to them.

The focus group (held near the end of the project) provided opportunities for two‐way reflection, between community partners (n = 6) and academics (n = 3) discussing what had worked well, and what could have been done differently. Two academics involved in the research chose to produce written reflections relating to their experiences of the engaged approach taken. Discussion and writing reflected on the process of engagement (why we got involved, experiences of involvement, logistical barriers and enablers), the impact this had on the study findings and the learning that can be taken from this experience.

To supplement our process of reflection, we also drew on feedback from participants in DeStress project learning and dissemination events. Specifically, evaluation forms from two training workshops held with sixty GPs and health professionals, and evaluation forms from the DeStress project conference (approx. 90 participants from academic, policy and health practitioner backgrounds) focusing in particular on feedback that related to the engaged research approach used in the project.

Two members of the research team collated the information gathered and shared a first draft of this paper with all the co‐authors. Quotes used in the paper were selected because they provided insightful perspectives from community partners or from particular disciplines/sectors. Permission was given for their use. Written and verbal feedback was generally positive with people feeling their perspectives and experiences had been fairly represented. Reviewer feedback and later drafts of the paper were also shared, and comments taken on board. All authors gave permission for their names to be used within this paper.

## 3 RESULTS

### 3.1 Initial motivations for involvement

Reflecting on the project enabled us to consider whether and how the engaged research approach used facilitated knowledge coproduction relating to poverty and mental distress. At the centre of this, was the necessity to understand why people had chosen to become involved in the research and the factors that supported or inhibited their engagement in the project over time. The research team were united by a motivation to understand the intersections between welfare reform and mental health provision in two locations where they had existing experience as researchers or as a health practitioner, and where discussions with community members had identified these issues as priorities for research. Community partners had mixed motivations for involvement, and some explained their initial scepticism around research involvement. Keith and Rachel, for example, explained.

I thought research projects were just research and that’s it, that they didn't *do* anything. So I thought what’s the point in it. Susanne (Community Connector) said it would eventually lead to GP training, but the reason for me was probably curiosity rather than changing anything at the time (Keith)

I didn't think we were going to be so involved. I thought you’d come and ask a few questions and that was it. Didn't think it would be as big as it was […] I thought it was going to be more using us as guinea pigs (Rachel)

Such comments are in part reflective of people's previous experiences with researchers and with negative interactions with a range of other service providers in which people in low‐income communities feel ‘done to’ rather than ‘engaged with’.^26^ They also point to the ways in which those working in a community‐facing role act as mediators, vouching for researchers’ credibility and consequently influencing recruitment and retention.^27^ Whilst the research team were committed from the outset to engaging with community partners, establishing how this might be done in practice initially felt daunting, with confidence only building over time as relationships developed. All authors agree that better communication and more time spent getting to know each other and discussing the aims of the research would have enabled a more open discussion around the potential of an engaged approach for knowledge coproduction. This may also have helped avoid reported feelings of ‘deflation’ amongst community partners after the first Advisory Board meeting was felt to be overly ‘academic’ and that people feared they would be ‘looked down on’. Karen, for example, reflected that.

Walking into a room of academics – if you’re like us, low‐income, depressed, got issues […] you automatically feel back‐footed as if you don't belong there and they are looking down on you (Karen)

Reflecting on this feedback researchers agreed there could have been more explicit discussion from the outset in early meetings about the value of co‐creation and what everyone was bringing to the group as individuals as well as within our academic roles, a point echoed by Gradinger et al^28^ who suggest that the values underpinning the involvement of people as partners in the research process are rarely made explicit despite the potential for these to impact its practice.

Community partners felt that dedicated time for team building (socializing with ‘just a name’ rather than a job‐description label) would have been a good exercise in the early stages, both to get to know each other as equals, and to explore the dynamics and role of the Advisory Board. More attention could also have been paid to the diverse backgrounds and pre‐existing relationships (or lack of) that community partners held with each other, and with various research team members. Debbie, a community partner who had not previously met the other community members, for example, explained how she felt quite isolated at early meetings because ‘we were there as community partners but we as a "community" didn't know each other.’

Community partners also initially felt they were being *defined by* and asked to participate solely *because of* their lived experiences of poverty‐related distress. However, as the project progressed, and relationships fostered, space was created where they felt empowered to move beyond ‘essentialized "service user" identities’^14^ to feeling that that their wider opinions were genuinely sought after and listened to. As Rachel commented, ‘we were not just asked about depression because we were depressed, but because we were part of the team’.

### 3.2 Sustaining engagement

A range of factors facilitated the on‐going engagement of community partners. The role of the Community Connector in liaising with community partners before every meeting or event helped to ensure they were accessible, with meeting times planned around childcare and work commitments, travel arrangements to get to and from meetings discussed and expenses met upfront and in cash. Rather than assume the ‘usual’ (academic) ways of communicating information about research meetings (email and sending out papers) supported involvement, the Connector asked individuals the best ways and times of sharing information and contacting them and responded with flexibility, for example ringing out of office hours or texting. Any emails were followed up with a phone call to discuss what the meeting was about and who would be attending, to ask whether there was anything they wanted to raise and ensure that practicalities were in place.

A flexible responsive approach was also important. For example, Advisory Board meetings were initially held in the geographically neutral space of the university, where technical facilities enabled the remote attendance of other Advisory Board members. However, following negative feedback from community partners about the power dynamics this space engendered, the next meeting was held in a community venue and was co‐chaired by the project lead (Felicity) and Debbie, one of the community partners. Discussions were held in small groups and then fed into the larger group, and a resident‐led tour of the neighbourhood took place after the meeting, enabling contextual insight of the area for all present. This shift in approach proved pivotal, as Debbie reflected, ‘Taking the meeting out of the stuffy academic environment and into a different space within our community did help change the power dynamics of the relationships. It helped us feel we were on a more equal footing, and really helped in the development of the work.’

Acknowledging the ways in which power dynamics may influence interactions draws attention to the danger of a tendency within health research to promote engagement solely for its contribution to ‘study success’,^29^ rather than from a rights‐based perspective that considers what makes involvement relevant for all concerned. In DeStress, flexibility in the research approach enabled community partners to identify and pursue project outputs that *they* felt were meaningful and important, alongside wider project requirements to produce more conventional, academically oriented outputs. To respect the time of research participants, for example, community partners felt it important to provide regular feedback on project progress for study site residents. On their suggestion, therefore, they worked alongside the Community Connector to develop project newsletters which were distributed to community venues and which included information about local support groups and services. Community partners also felt it important to create an educational resource to support patients who may be struggling to access help, resulting in them producing posters with key messages for patients that are now available for display in waiting rooms and community settings.^30^

### 3.3 How did coproduction affect research findings?

The diverse perspectives offered by the Advisory Board enabled us to elicit a range of interpretations of the data and to reflect on the analytical lens (researcher, clinician, community partner) through which the interpretation was being made. For example, interview and focus group data suggested that fear of being judged as a parent influenced women's decisions to avoid discussion of mental health with health practitioners. This finding resonated strongly with community partners, who then gave examples from their own and others’ lived experience. These discussions provided the research team with a much deeper understanding of the ways that fear around child protection procedures can significantly impact on the choices that parents make about seeking mental health support.

Opportunities to question and validate the data were also important when analysing the anonymized videos and transcripts of GP consultations. Here, the academics, following theoretical conventions around good clinical communication gained from published evidence in Conversation Analysis techniques, were highly critical of the practice of a GP who appeared to miss many of the cues given by the patient about how social isolation and economic hardship were impacting on her mental health. However, community partners were much more positive about this consultation, giving considerable weight to the warmth shown by the GP when he hugged the distressed patient—an action they interpreted as both empathetic and legitimizing of the patient's concerns. What the research team had seen as ‘good’ and ‘credible’ evidence from scientific literature was therefore challenged. This, in turn, significantly impacted subsequent data analysis and write‐up, with much more emphasis placed on the relational aspects of health consultations, including the feelings and concerns held by low‐income patients before and within a clinical encounter; the possible assumptions being made by both patient and doctor about each other's backgrounds and attitudes; and the importance of striking an appropriate non‐judgemental tone in data analysis. Such instances helped build confidence amongst community partners to challenge academic ideas, and helped foster a bi‐directional relationship in which both academic and experiential forms of knowledge were seen as valuable.^31^ One community partner, for example, commented that involvement in the project ‘has given me the confidence to become involved in various other projects and has given me the confidence to give presentations to academics and people from all walks of life’.

Co‐analysis between researchers and community partners also ensured greater attention was given to considering a patient's trajectory beyond the confines of the consultation, including how difficult, both practically and emotionally, they may have found it to make and keep the appointment, how they might feel afterwards, and how this might affect adherence to any treatment recommendations. Recognizing the relational aspects of the consultation and the broader socio‐economic context within which health‐related decisions were being made by low‐income patients thus enabled us to move beyond simplistic framings around mental health access inequities to make recommendations that recognized the complex intersections between poverty‐related distress, welfare reforms, medicalization, and the pressures facing GPs.

### 3.4 Coproduction and dissemination of outputs

Recommendations from research findings were used to inform the development of guidelines for health practitioners supporting patients from low‐income backgrounds. A working group comprising a community partner (Debbie), researcher (Felicity) and a GP trainee met regularly at a community venue to develop the written resources. To help communicate the patient's perspective, a community partner with film‐making expertise (Keith) was commissioned to make short films with local people discussing their experiences and thoughts about mental distress and seeking help.

The dynamics of the working group responsible for co‐creating GP training materials were significant because the specific focus of the task—informing and changing GP practice—itself highlighted differences in the expectations and underlying assumptions of both GPs and patients. For example, patients are feeling that GPs do not understand their circumstances and perspectives, and GPs are feeling that patients do not understand the working practices and systems that they are constrained by. Challenging the assumptions that GPs can make about people living in difficult circumstances and emphasizing the work required for GPs during consultations to demonstrate that they are aligned *with*, rather than making judgements *on*, people from low‐income neighbourhoods became an important component of the training materials, alongside changing patient expectations about the types of response a GP might realistically provide.

The draft training materials were trialled at workshops with GP trainees and with established GPs. Community partners were involved in planning and delivering the workshops alongside the researchers, and provided critical feedback in response to questions raised by workshop participants, again providing opportunities to challenge preconceptions held by academics and health professionals. For example, a community partner upset by a participant's comment about the possible need for parenting courses to be provided for low‐income patients raising mental health concerns was able to challenge the comment and explain why this kind of reaction might alienate parents fearful of statutory involvement from seeking mental health support.

Towards the end of the project, we held a two‐day conference in London, with one day aimed at academics and the second targeting policymakers and health practitioners. Community partners were involved in conference planning and delivery, giving presentations and participating in panel discussions.^25^ Academics and health practitioners remarked in their conference evaluation forms that the community partners’ level of involvement was an unusual and welcomed element of the event. Listening first‐hand to people's lived experiences challenged their assumptions and increased their understanding of, and receptivity to, key messages around patient's lives, expectations and concerns. A number of attendees commented how these presentations made them reflect on their own practice; one attendee explained how,

They [community members] bring you up close to see how important it is to get it right, and also the potential for harm ‐ that people can have such a negative experience that it will prevent them from seeking help in the future.

Two community partners also chose to co‐present findings with research team members at other events—Karen spoke at a national health inequalities conference on the role the community partners were playing in shaping the research, whilst Debbie co‐led a workshop at an international clinical conference on the lived experiences of low‐income patients attending GP consultations for mental health.

### 3.5 What did involvement mean?

Reflecting on their involvement in the study, Karen and Debbie felt that it was their growing sense of the project's potential to make an impact on practice and policy that sustained their motivation,

I knew it was an important subject […] the thing that would’ve really put me off was if it was just research for research sake – shoved in the bottom of a cupboard ‐ but it sounds like it’s getting out there in the wider public where it can help and influence people (Karen)

I believed that this project wasn’t just a project, it has a life after and will mean a lot to people. If I thought it was a waste of time, I can guarantee I’d have said "no thank you," very early doors (Debbie)

Whilst some community partners initially undervalued the insights they contributed as ‘just common sense’, they increasingly appreciated the importance of their input. This was especially evident in discussions around GP‐patient interactions, where community partners challenged some of the assumptions held by health professionals. Spending time listening to patients outside of the consultation room and in a setting in which all were considered experts provided opportunities for health professionals to reflect on their assumed knowledge, and to question the appropriateness of current responses to poverty‐related distress. Richard, the GP on the research team, for example, explained how the project's engagement with community partners made him more aware of how his patients may be feeling when they come into a consultation and described his involvement in the study as an ‘important part of a general reorientation of how I think medicine should be practiced’. Similar reflections were found in the written evaluations captured following the GP workshops, with the most common comment relating to an increased awareness to listen, be aware of presumptions and bias and to show empathy with their patient. As well as recognizing the broader socio‐economic context in which low‐income patients sought mental health support, GPs also reported that they would be less inclined to rush to prescribe medications for distress that was inherently social/structural in nature, and to reassess current long‐term prescriptions when treatments were not of obvious benefit to their patient.

Academics within the team described how working alongside community partners helped shape their own thinking beyond the project. Rose, a psychologist, for example, reflected on how their contributions had demonstrated the potentially powerful role played by community groups in providing mental health peer support. The conceptualizing of mental health as ‘everyone's responsibility’, or a societal issue rather than one which can be responded to by health professionals alone became one of the project's key messages, alongside the need to re‐orientate thinking from a narrow medical model of treatment to a broader bio‐psychosocial model.

## 4 DISCUSSION

Reflecting on the research process, we collectively identified a number of principles to inform future engaged work. Whilst most of these principles reflect recognized good practice in engaged research amongst a broad population group, we have sought to draw out aspects of our experiences that are of especial relevance to engaging low‐income communities—a seldom‐heard population—in mental health research.

### 4.1 Developing trusting relationships

Engagement needs to be built on trust and mutual respect—factors particularly vital when working with communities that are systematically marginalized and often unfairly and blatantly judged within current political and popular rhetoric.^32^ In DeStress, the Community Connector acted as ‘bridge’ and ‘interpreter’ between the research and the community. Existing relationships within both study sites, and dedicated time to support the engagement of community partners, was deemed crucial to the project's success. Community partners commented on the importance of this role being undertaken by the ‘right’ person who is trusted, respected and visible, who understands the dynamics of working in a low‐income community and can operate flexibly, whilst researchers recognized the importance of this person being able and willing to negotiate routes through university structures to support involvement. Whilst such roles are recognized as being important in supporting engaged research,^33^ ensuring that it undertaken by someone already known and trusted may also be crucial to community partners becoming involved in the first place.

### 4.2 Negotiating language

The negotiation of accessible and sensitive language was a vital element of the DeStress project. Using non‐judgemental and non‐clinical terms was central in enabling researchers and community partners to identify with experiences rather than with labels that served to perpetuate unhelpful stereotypes. On the advice of the community partners, researchers used terms such as ‘stress’ and ‘feeling low’ in relation to mental health. Understanding the potentially pejorative impact of clinical terms such as ‘depression’, ‘anxiety’ or ‘mental health’ (unless used by research participants) was pertinent given the nature of the research questions being investigated and allowed us to work together to explore the medicalization of poverty‐related distress by paying attention to the ways in which people described and made sense of their own experience—in turn creating space for us to question the value of clinical diagnosis and medicalization within such situations. Similarly, the need to use terms such as ‘low income’ rather than ‘poverty’ was underlined by community partners who felt strongly that poverty is relative and commented that they did not see themselves as ‘poor’ or ‘deprived’.

### 4.3 Structural considerations

Traditional academic practices and timeframes often operate as barriers to inclusivity and engagement in research^34^ and present administrative obstacles to the flexible and responsive approach critical to engagement. Small but important details such as availability of cash expenses *before* an event, or the creation of an accessible website, required a significant amount of academic staff time to try to make organizational systems support engaged research. Our experience suggests that a research project with a genuine commitment to engagement must take into account the extra time needed to navigate these systems. It also supports arguments for researchers to put pressure on institutions and funders to examine how their systems could better support engaged research.^35^

### 4.4 Recognizing motivations for involvement

‘Making a difference’ was the main driver sustaining community partners’ involvement, making it vital that research teams recognize what this actually means for non‐academic partners, and that appropriate opportunities are put in place for these visions to be realized. In DeStress this was in part addressed through supporting the production of community newsletters and training materials. However, within her role, Susanne also recognized the importance of the community partners and their wider community groups developing relationships with each other. Hence, she organized visits between groups, as well as supporting their on‐going initiatives. We are all conscious that this could have been taken further and that with appropriate resourcing the Connector role could also have facilitated links between these groups and larger organizations involved in the project, for example public health and third sector organizations.

### 4.5 Relational working

Literature on engagement frequently overlooks the intricacies of relationships amongst those involved in research and knowledge co‐creation.^36^ In the initial stages of the project, community partners felt that ‘we were probably defined by what we do rather than who we are’ (ie resident or researcher), and that this impacted negatively on them when they felt they did not bring ‘professional’ expertise to the table. Alongside this, the danger of those involved in research becoming an uncritically officialized ‘community’ voice is well known and also risks homogenizing the experiences of what are in reality, likely to be diverse experiences and perspectives.^37^ In DeStress, community partners were experiencing quite different personal situations and challenges—as were the academics. Spending informal time together (eg socializing over lunch after meetings) proved enormously important because it provided opportunity to ‘get to know *people as people*’, share stories and experiences on a more equal footing and recognize the diversity of perspectives that were held both within the research team and amongst the community partners.

### 4.6 Allowing time for coproduction

Successful engagement and relationship building requires dedicated time.^38^ Whilst academics tend to start projects ‘research ready’, a process of familiarization on the research process, as well as its potential and limitations can be important for those outside of academia. In DeStress, time was allocated for the Connector and main researcher to build and sustain networks within the study sites – and it was recognized that community partners have other commitments to work and family that take priority and require flexibility. However, the different roles and responsibilities of the academics meant that time available for engagement varied widely, which could, at times, leave some feeling isolated from the process.

The longevity of the project was crucial to building trust and allowing a shared understanding of the research. However, commenting at the end of the study, Rachel asked ‘what's the next project, we are ready to start now!’, suggesting she felt familiar with the processes and structures of research at the end in a way the academics had not paid sufficient attention to at the beginning. This highlights a tension between the time‐limited nature of most research projects and funder's timescales, and the importance of time to create the conditions for truly engaged research. It also raises important questions around endings that need full consideration in future research. Whilst community partners and academics were aware of, and worked towards, the project's end date, the applied nature of the findings has meant that impact and dissemination activities continue ‐ however we share frustration that resources to support our involvement are now very limited as the project funding has ended.

## 5 LIMITATIONS

In this paper we have attempted to present an honest critique of our methodology to promote transparency and to maximize learning opportunities from our work about coproducing knowledge on mental health through an engaged approach within low‐income communities. We are aware that in reflecting on our approach we (perhaps in particular, the academics) may be more attuned to hearing positive aspects of the engagement. However, aware that publications on engagement often focus too heavily on disseminating positive experiences,^35^ we feel that our attempts to candidly reflect on what we felt did and did not go well are integral to ethical research practice.^39^

We are aware that the reflections presented here may not fall in line with academic conventions that dictate the scientific robustness of research publications. However, as authors from very diverse backgrounds, we agree that this paper is reflective of our experiences, and what we feel is important to share in an accessible manner with others from within and beyond academia who are thinking of undertaking, or being involved in, similar projects.

## 6 CONCLUSIONS

An engaged research approach meant that the project was successful in giving centrality to the voices of those affected by poverty‐related mental distress. The findings of the project and the implications for patients and GPs, including the ways in which these findings are being disseminated and used in training materials, have been co‐created through an evolving process of academics and community partners learning together.

Working together, we have sought to show how this has affected the study and what we have all learnt from the process, both to share our learning and, through setting out core principles to inform future research, to advocate this approach to engaging communities who traditionally do not participate in research, either as participants or as partners. We did not seek to assess the ‘value’ of this approach in an instrumental, causal way, but have tried to show the very real impact that involving community partners has had on the reconceptualization of mental health and the implications of this for diagnosis and treatment in the context of poverty‐related distress. That we are all developing ideas for future research to undertake together feels like a very positive and additional outcome from this work.

## CONFLICT OF INTEREST

The authors declare that there is no conflict of interest.

## Data Availability

Anonymized focus group and interview transcripts from participants who consented to data sharing, plus additional supporting information, are available from the UK Data Service, subject to registration and permission. Details of how to request access are available from the UK Data Service at: http://doi.org/10.5255/UKDA‐SN‐853788
